# Energetic context determines the effects of multiple upwelling-associated stressors on sea urchin performance

**DOI:** 10.1038/s41598-021-90608-6

**Published:** 2021-05-31

**Authors:** Kindall A. Murie, Paul E. Bourdeau

**Affiliations:** 1 Telonicher Marine Laboratory, Humboldt State University, Trinidad, USA; 2 Department of Biological Sciences, Humboldt State University, Arcata, USA; 3 Present Address: Department of Biology, University of Washington, Seattle, USA

**Keywords:** Climate change, Marine biology, Climate-change ecology, Community ecology

## Abstract

Globally, kelp forests are threatened by multiple stressors, including increasing grazing by sea urchins. With coastal upwelling predicted to increase in intensity and duration in the future, understanding whether kelp forest and urchin barren urchins are differentially affected by upwelling-related stressors will give insight into how future conditions may affect the transition between kelp forests and barrens. We assessed how current and future-predicted changes in the duration and magnitude of upwelling-associated stressors (low pH, dissolved oxygen, and temperature) affected the performance of purple sea urchins (*Strongylocentrotus purpuratus*) sourced from rapidly-declining bull kelp (*Nereocystis leutkeana*) forests and nearby barrens and maintained on habitat-specific diets. Kelp forest urchins were of superior condition to barrens urchins, with ~ 6–9 times more gonad per body mass. Grazing and condition in kelp forest urchins were more negatively affected by distant-future and extreme upwelling conditions, whereas grazing and survival in urchins from barrens were sensitive to both current-day and all future-predicted upwelling, and to increases in acidity, hypoxia, and temperature regardless of upwelling. We conclude that urchin barren urchins are more susceptible to increases in the magnitude and duration of upwelling-related stressors than kelp forest urchins. These findings have important implications for urchin population dynamics and their interaction with kelp.

## Introduction

**Figure 1:**
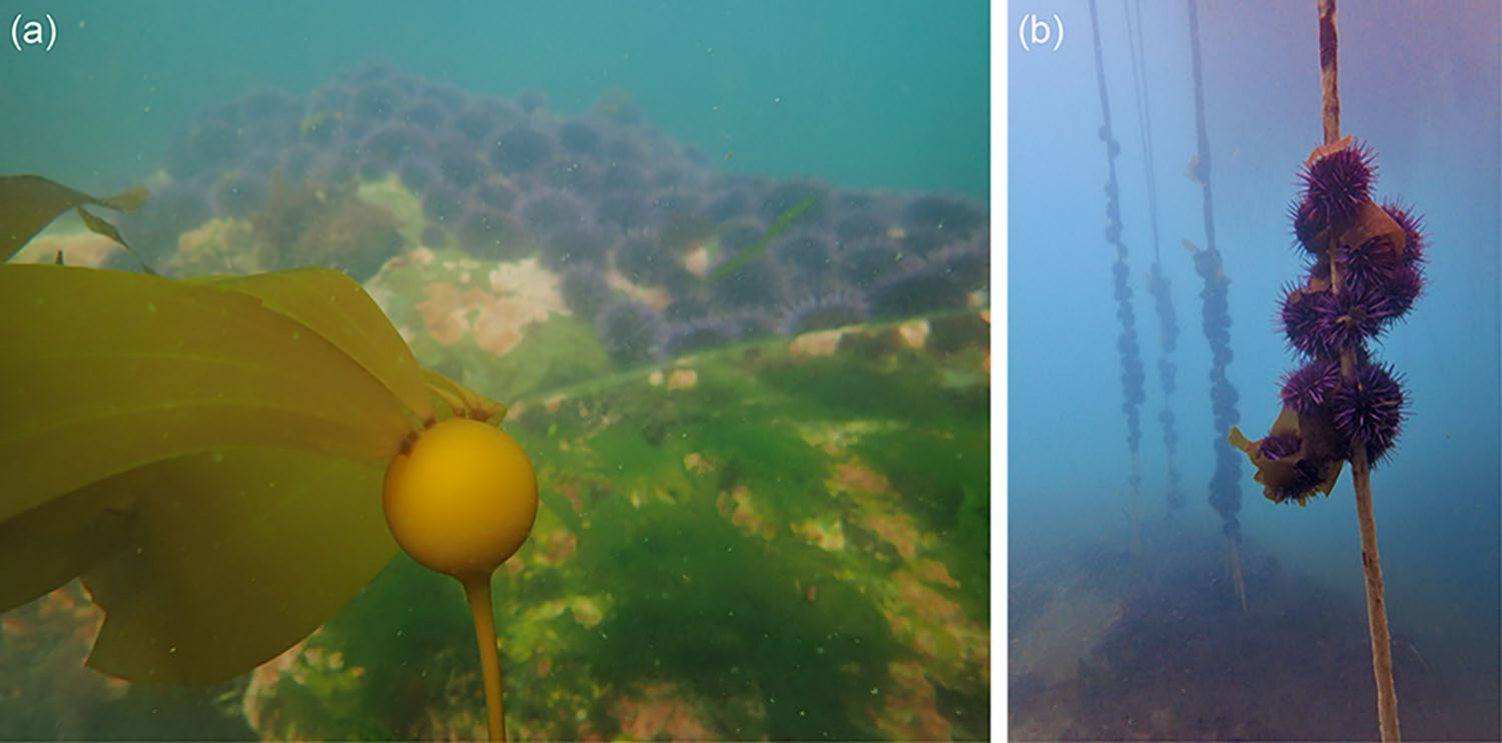
(**a**) A young bull kelp (*Nereocystis leutkeana*) recruit (foreground) and a front of actively grazing purple sea urchins (*Strongylocentrotus purpuratus*) (background); and (**b**) purple sea urchins actively climbing and consuming the stipes of bull kelp in a rapidly declining kelp forest fragment in northern California. Photo credit: K. Murie.

In many regions of the world, kelp forests are in decline due to climate change, overfishing, and direct harvest, but in others, kelp abundance is stable or increasing, indicating the dominance of regional drivers or region-specific responses to global drivers of change^1^. In some regions, kelp decline can be attributed to sea urchins, the major grazers of kelp worldwide^2–5^. Disruption to the interplay between sea urchin grazing and kelp forest productivity can tip the balance between stable states that alternate between diverse kelp forests and species-depauperate urchin barrens and the transition from one state to the other can be initiated by several factors, including the abundance of algal food, predators, storm intensities, and incidence of disease^3, 6^. For example, when kelp forests are productive and healthy, sea urchins remain relatively immobile, preferring to feed on the drift kelp that is produced and sloughed off in large quantities by standing kelp^7, 8^. However, when large-scale disturbance (e.g., winter storms or El Niño events) remove large amounts of standing kelp biomass, sea urchins mobilize and graze on young kelp recruits (Fig. 1a) and actively climb and consume the stipes of adult kelp (Fig. 1b), removing remaining kelp and preventing the reestablishment of the forest^6^.

Further, recruitment peaks can cause sea urchin densities to spike^9^, resulting in intense grazing that can transform kelp forests into urchin barrens^10^. In the California Current Large Marine Ecosystem (CCLME) a ‘perfect storm’ of multiple factors has led to historic increases in purple sea urchin (*Strongylocentrotus purpuratus*) abundance and dramatic reductions (> 90% loss in canopy cover) in bull kelp (*Nereocystis leutkeana*) forests along the northern California coast, many of which have been converted to urchin barrens^5^. The kelp loss has had cascading and detrimental effects on other organisms and the vital ecosystem services they provide^11^.

Because urchins can play a major role in the destruction of kelp forests^4, 5^, it is critical that we gain a greater understanding of how current and future environmental conditions will shape the performance of this key grazer. One way that sea urchins and their interaction with kelp may be affected is via coastal upwelling, a process that brings cold, nutrient-rich, hypoxic and acidic water to nearshore coastal environments seasonally. Upwelling thus simultaneously changes multiple environmental factors that can affect species performance and their interactions with other species^12^. For example, slight changes in water temperature associated with upwelling have been shown to dramatically alter the consumptive effects of predatory sea stars (*Pisaster ochraceus*) on rocky shore mussels (*Mytilus californianus*). Likewise, in estuarine food webs, low, but nonlethal, dissolved oxygen (DO) concentrations can modulate the consumption rates of predatory invertebrates on fish eggs and larvae^13^. Further, in some regions, present-day upwelling can deliver water as acidic as that predicted for global ocean averages 50–100 years from now^14^, conditions that can alter competitive interactions between turf algae and kelp, shifting rocky reefs to kelp-dominated habitats^15^. Alternatively, upwelling-driven increases in nutrients can lead to increases in algal primary productivity, that can outpace consumption by grazers^16^.

Whereas the majority of previous studies have focused on separating out the effects of individual stressors associated with upwelling on species performance^17, 18^, few have examined the effects of these stressors in concert^19^. Even fewer have examined how future predicted and temporal variability in these stressors will influence species interactions (but see^20, 21^). However, upwelling in coastal systems is an inherently multi-stressor phenomenon with highly correlated changes in temperature, DO, and pH occurring intermittently, over scales of days to weeks^22, 23^. Thus, to understand how current and future upwelling conditions will affect key species interactions, it is necessary to examine multiple stressors acting concurrently under natural temporal scales of variability.

Upwelling plays a defining role in the biogeography, ecology, and productivity of the CCLME^24–26^, dominating during the spring and early summer months and dissipating in the late summer and fall, before the start of winter storms^27^. The magnitude of current day upwelling conditions varies regionally along the CCLME^25^. Off the coast of southern Oregon and northern California, between Cape Blanco and Cape Mendocino, upwelling can last as few as three days and as long as 12, with high variability^28^. On average, upwelling periods are shorter than non-upwelling periods in this region and in 2018, Trinidad Harbor (located roughly midway between Capes Blanco and Mendocino) experienced pH levels below 7.8 49% of the time, DO below 8.0 mg L^−1^ 44% of the time, and temperature below 10 °C 23% of the time, during upwelling season^29^. Although greenhouse warning has been proposed to intensify alongshore wind stress on the ocean surface, leading to acceleration of coastal upwelling^30^, subsequent analyses have not reached consensus on historical and projected trends in coastal upwelling^31–34^. Despite this, most models and historical trends predict that nearshore coastal regions in northern California will experience either more frequent^35^, prolonged^36^, or intense^37, 38^ periods of upwelling. For example, Iles et al.^28^ showed that annual mean duration of upwelling increased by 26–86% from 1967 to 2010 in regions of Oregon and northern California, and recent models predict that upwelling will increase in this region (^38–40^; but see^41^). Along with changes in the duration of upwelling, current models predict that by 2100, global ocean pH will decrease to 7.7^42^, DO will decrease between 0.6 and 2.0% from current levels^42^, and sea surface temperatures will increase by 2–3 °C^42^ under current anthropogenic climate change^42–44^. Further, anomalous marine heat waves have already become more frequent, longer lasting, and more intense in the past few decades^45, 46^. Taken together, coastal habitats along the CCLME are therefore predicted to experience an increase in the duration and magnitude of stressors associated with coastal upwelling and marine heat waves, with potentially important consequences for kelp-urchin interactions.

With the deforestation of kelp forests in many regions of the world, it is crucial to begin to predict how both current and future upwelling will impact the performance of sea urchins, arguably one of the largest threats to kelp globally. Further, the rapid decline in kelp forest habitat and concomitant increase in urchin barrens in northern California provides a unique opportunity to test whether organismal condition may play a role in mediating the effects of upwelling stressors on their performance. Because the negative effects of environmental stressors on organisms often result from energetic deficiencies due to increased metabolic demand (i.e., an energetic tradeoff^47, 48^), several studies have hypothesized, and found, that these negative effects can be reduced in situations where energy (e.g., food) is more readily available^49–51^. Kelp forest and urchin barrens urchins provide an excellent model to test this hypothesis. In kelp forests, high-quality food is abundant and sea urchins are well-nourished, moving little and feeding on the drift kelp. In urchin barrens, drift kelp is sparse, and sea urchins are poorly nourished, actively grazing the substratum for poor-quality food. The high densities of urchins and lack of kelp food resources in urchin barrens can result in urchins in poorer condition and with reduced gonad production (indicative of an energetic tradeoff) relative to kelp forest urchins^52^. We therefore hypothesized that better-condition kelp forest urchins would be less negatively affected by upwelling-related stressors than poorer-condition barrens urchins.

We tested this hypothesis in a 4-week mesocosm experiment, where we manipulated both the duration and strength of upwelling events—pulses of low dissolved oxygen, pH, and temperature that can occur during the upwelling season in the CCLME-to assess the direct effects of present-day and future-predicted upwelling-associated stressors (low pH, dissolved oxygen, and temperature) on the grazing rates, survival, and gonad development of purple sea urchins from kelp forest and urchin barren habitats. By designing a mesocosm experiment that provides a realistic, temporally variable, and multiple-stressor habitat-specific context, we aimed to provide better insight into how purple sea urchins, bull kelp’s major grazer, will be affected under current day and future climate scenarios.

## Results

### Initial sea urchin condition and experimental conditions

Larger kelp forest urchins had proportionally more gonads than urchin barren urchins of a similar size (ANCOVA, *F*_3,26_ = 19.32, *P* < 0.0001; see Supplementary Fig. S1 online). The CO_2_ dosing system succeeded in achieving the pH conditions very close to the targets for each upwelling severity treatment (Table 1; also see Supplementary Fig. S2 online).

**Table 1:** Desired, achieved, and associated carbonate chemistry parameters for each treatment in the mesocosm experiment.

Treatment	Upwelling condition	Duration	Desired conditions			Achieved conditions			Total carbonate parameters			
			pH	DO (mg L^−1^)	Temp (°C)	pH	DO (mg L^−1^)	Temp (°C)	*p*CO_2_ (μatm)	TA (μmol kg^−1^)	Ω calcite	Ω aragonite
Current day	Nonupwelling	11	7.9	Equilibrium	12	7.87 (0.02)	9.28 (0.12)	11.7 (0.39)	696.64	1994	1.67	1.06
Current day	Upwelling	4	7.6	5	8	7.58 (0.03)	4.96 (0.20)	8.2 (0.55)	1233.57	1953	0.86	0.54
Future 1	Nonupwelling	10	7.8	Equilibrium	14	7.81 (0.05)	8.73 (0.12)	13.7 (0.42)	685.02	2015	2.01	1.29
Future 1	Upwelling	5	7.4	4	9	7.41 (0.04)	4.01 (0.17)	9.4 (0.55)	1840.61	1985	0.82	0.52
Future 2	Nonupwelling	7	7.7	Equilibrium	16	7.71 (0.03)	8.61 (0.15)	15.9 (0.21)	812.92	2017	1.63	1.04
Future 2	Upwelling	8	7.3	3	10	7.29 (0.07)	3.00 (0.31)	10.4 (0.65)	1943.07	1981	0.58	0.37
Future 3	Nonupwelling	5	7.6	Equilibrium	18	7.63 (0.06)	8.27 (0.44)	17.6 (0.60)	951.89	2084	1.79	1.15
Future 3	Upwelling	10	7.2	2	11	7.25 (0.05)	2.09 (0.39)	11.0 (0.52)	2020.91	1991	0.79	0.50

Achieved conditions are hourly averages and numbers in parentheses are standard deviations.

### Sea urchin grazing

**Figure 2:**
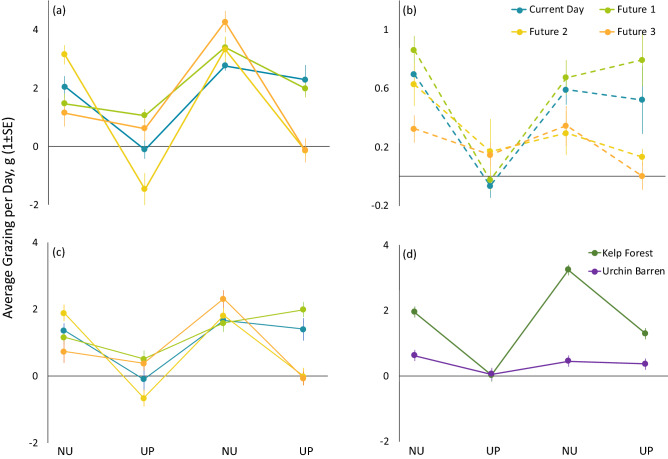
Average (1 ± SE) grazing rates through time (during each non-upwelling and upwelling event) for (**a**) kelp forest and (**b**) urchin barren urchins, and (**c**) urchins pooled across habitat in each of the four upwelling severity treatments; and (**d**) kelp forest and urchin barren urchins pooled across upwelling severity treatments. KEY: *NU* non-upwelling event, *UP* upwelling event.

There was a three-way interaction between upwelling severity treatment, urchin habitat, and time (upwelling event) on urchin grazing rates (Table 2), indicating that the interactive effects of treatment and time (upwelling event) were different for kelp forest (see Supplementary Table 1a online) and urchin barrens urchins (Fig. 2b).

**Table 2:** Results of linear mixed-effects model fit by REML on sea urchin grazing in mesocosm experiment.

Tank	Intercept	Residual			
*Random effects*					
Standard deviation	0.213	1.73			

	Estimate	SE	*df*	*t*	*P*
*Fixed effects*					
Intercept	0.689	0.552	712	1.25	0.212
Treatment	0.316	0.209	18	1.51	0.149
Habitat	− 0.020	0.762	712	− 0.026	0.979
**Time**	**0.811**	**0.203**	**712**	**4.00**	**< 0.001**
Treatment** × **habitat	− 0.369	0.290	712	− 1.27	0.203
**Treatment × time**	− **0.289**	**0.074**	**712**	− **3.92**	**< 0.001**
**Habitat × time**	− **0.767**	**0.287**	**712**	− **2.68**	**0.008**
**Treatment × habitat × time**	**0.252**	**0.104**	**712**	**2.42**	**0.016**

Bold text indicates statistical significance at α = 0.05.

During initial non-upwelling conditions, kelp forest urchins grazed at similar rates in all treatments except in Future 2, where urchins grazed almost 3 × faster than urchins in Future 1 and 3 (Fig. 2a, see Supplementary Table 1a online) and Current Day urchins grazed at rates intermediate to Future 2 and Future 1 and 3. During exposure to the first upwelling event, grazing by both Current Day and Future 2 urchins was reduced to near zero, but there was no effect on Future 1 and Future 3 urchins, whose grazing remained similar to initial non-upwelling levels (Fig. 2a, see Supplementary Table 1a online). Kelp forest urchins in all treatments increased their grazing rates relative to the prior upwelling event after the respective header tanks returned to non-upwelling conditions, returning to (Current Day, Future 2), or exceeding (Future 1, Future 3) initial non-upwelling grazing rates (Fig. 2a, see Supplementary Table 1a online). During the second and final upwelling event, grazing rates by kelp forest urchins in Future 2 and 3 treatments were reduced to near zero, whereas urchins in Current Day and Future 1 treatment maintained grazing rates similar to those during the previous non-upwelling event (Fig. 2a, see Supplementary Table 1a online).

During initial non-upwelling conditions, urchin barren urchins in Current Day, Future 1, and Future 2 treatments grazed at similar rates, with urchins in Future 3 grazing at roughly half the rate. Upon exposure to the first upwelling event, grazing by urchins in Current Day and Future 1 was reduced to near zero. Although there was no statistically detectable effect of the first upwelling event on Future 2 and Future 3 urchin grazing (see Supplementary Table 1a online), there was an observable downward trend (Fig. 2b). Just as with kelp forest urchins in the same treatments, urchin barren urchin grazing in Current Day and Future 1 rebounded to pre-upwelling exposure grazing levels during the second non-upwelling event, and remained at similar levels during exposure to the second upwelling event (Fig. 2b, see Supplementary Table 1a online). Grazing by barrens urchins in Future 2 and Future 3 treatments during the second non-upwelling event were relatively lower than those in Current Day and Future 1 treatments and similar to those during the previous upwelling event, trending further downward in the second and final upwelling event (Fig. 2b, see Supplementary Table 1a online).

When averaged across urchin habitat, upwelling treatment effects depended on upwelling event (Fig. 2c, see Supplementary Table 1a online). Urchins in Current Day and Future 2 treatments showed a general trend toward reduced grazing during exposure to the first upwelling event, followed by a return to pre-upwelling levels of grazing during the second non-upwelling event (Fig. 2c, see Supplementary Table 1b online). Urchins in the Future 1 treatment were better able to maintain their grazing rates between initial non-upwelling conditions and the first upwelling event, although they increased their grazing rates threefold overall in the second non-upwelling event and maintained them during the second upwelling event (Fig. 2c, see Supplementary Table 1b online). Urchins in the Future 3 treatment showed a sixfold increase in grazing rates during the second non-upwelling event, but in contrast to Future 1 urchins, their grazing rates dropped sharply to near zero after exposure to the second upwelling (Fig. 2c, see Supplementary Table 1b online).

Across upwelling severity treatments, kelp forest urchin grazing rates were 3–7 × higher than barrens urchins during non-upwelling events (Fig. 2d, see Supplementary Table 1c online). However, during the first upwelling event, kelp forest urchin grazing was reduced to near zero, similar to that of barrens urchins (Fig. 2d, see Supplementary Table 1c online). Kelp forest urchin grazing rebounded during the second non-upwelling event, to levels more than 1.5 × higher than during initial non-upwelling conditions (Fig. 2d, see Supplementary Table 1c online). Further, although kelp forest urchin grazing was reduced by a factor of 2.5 upon exposure to the second upwelling event, it did not dip to rates as low as during the first upwelling event. Urchin barren grazing remained similarly low after exposure to the first upwelling event and throughout the duration of the experiment (Fig. 2d, see Supplementary Table 1c online).

### Sea urchin survival and gonad development

**Figure 3:**
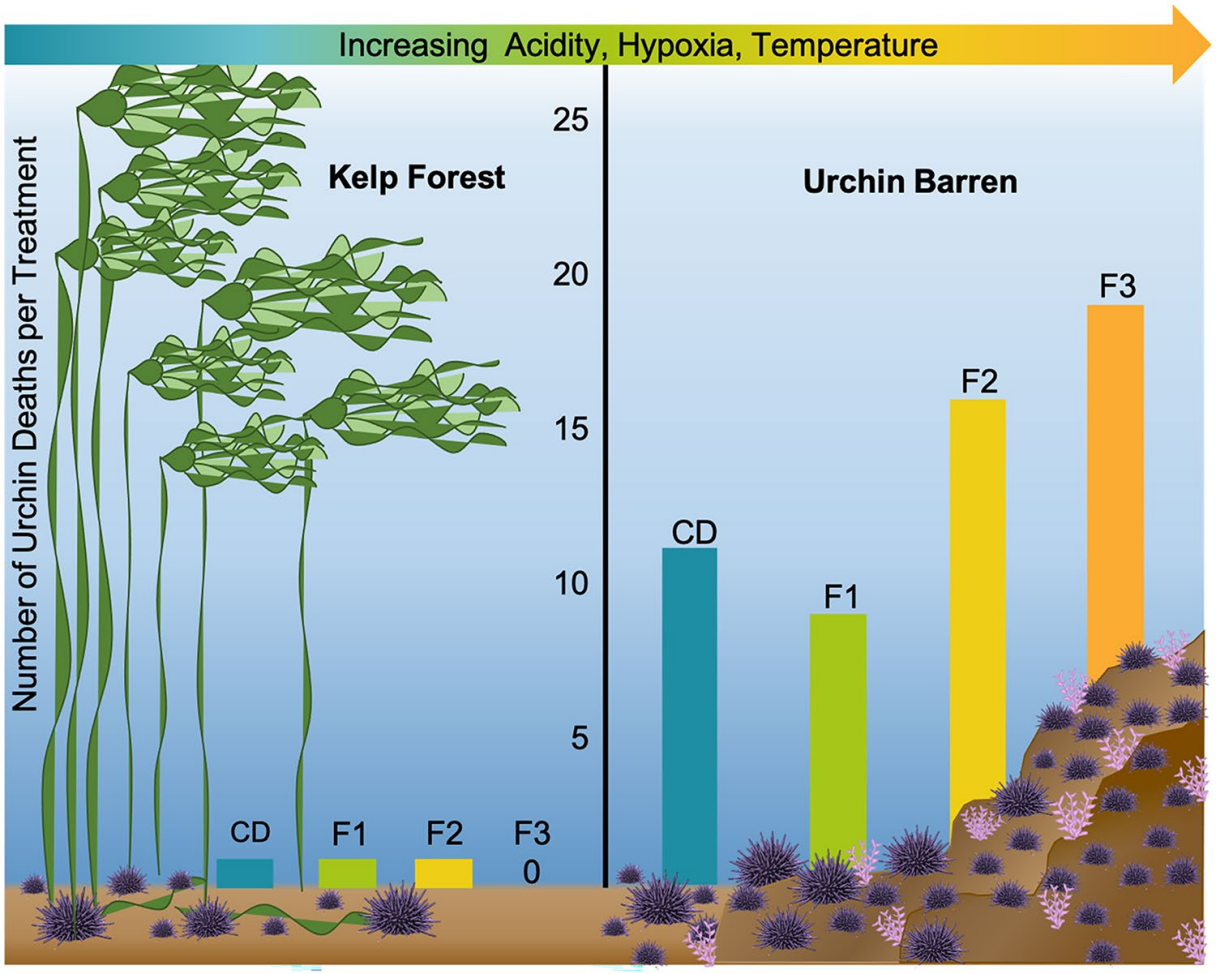
Total number of deaths for kelp forest and urchin barrens urchins in each of the four upwelling severity treatments. KEY: CD - Current Day, F1 - Future 1, F2 - Future 2, F3 - Future 3.

Upwelling severity had no effect on urchin mortality (z = 1.26, *df* = 36, *P* = 0.208), but urchin habitat did (z = 4.91, *df* = 36, *P* < 0.001), with urchin barren urchins suffering ~ 18 times (55 to 3) the mortality of kelp forest urchins (Fig. 3).

**Figure 4:**
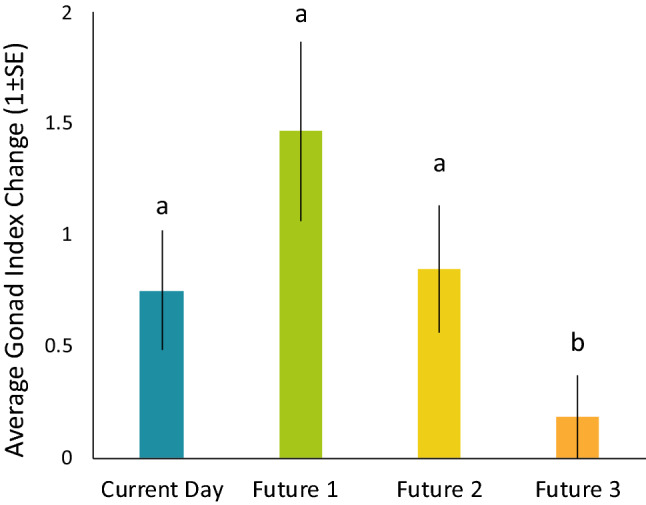
Average (1 ± SE) gonad index change for kelp forest urchins in each of the four upwelling severity treatments.

High mortality in barrens urchins led to only four urchins surviving the duration of the experiment, thus treatment effects on the gonad indices of barrens urchins could not be assessed. Upwelling severity did affect the condition of kelp forest urchins that survived the duration of the experiment (ANOVA, *F*_3,26_ = 2.89, *P* = 0.05; Fig. 4), with kelp forest urchins in the Future 3 treatment with 59–76% lower gonad indices than those in any of the other upwelling severity treatments (Tukey’s HSD, all *P*’s > 0.330).

## Discussion

Our results indicate that purple sea urchins (*S. purpuratus*) from different habitats (kelp forests and urchin barrens) and maintained on habitat-specific diets exhibit differential susceptibility to both current day upwelling, and future-predicted increases in the magnitude and duration of abiotic stressors associated with upwelling. Kelp forest urchins, which were more resilient, survived well under all upwelling severity treatments. This was in sharp contrast to urchin barren urchin mortality, which was high across all upwelling severity treatments. These results predict potentially important population- and ecosystem-level consequences, as kelp forest urchins, so long as they have abundant and high-quality kelp food resources, will be more resilient to current- and near-future upwelling stress than urchin barren urchins limited to poorer quality algal diets. Although there is currently an overabundance of purple sea urchins on the northern California coast because of low mortality for certain age classes, owing to a lack of natural predators and anomalously strong recruitment^5^, our results suggest that as urchin barrens become more common in these areas, purple urchins may experience increasingly higher rates of mortality owing to increased exposure to and magnitude of upwelling stress.

We hypothesize that mortality differences in kelp forest and urchin barren urchins were due to in part to the effects of diet during the study and to the condition status previous to the study. Our goal was not to separate out whether initial urchin condition (which is a function of food quality in their source habitats), or food quality in the experiment (which was intended to maintain condition differences throughout the experiment) was responsible for condition-dependent sensitivity to upwelling-stressors. Rather our goal was to determine whether poor-condition urchins with a low-quality food source, as is the case in urchin barrens habitats, would be more susceptible to upwelling-associated stressors than good-condition urchins with a high-quality food source, as is the case in kelp forests. In urchin barrens, high quality food is scarce or absent, and urchins must rely on poor-quality coralline algae as opposed to in healthy bull kelp forest habitats, where abundant *Nereocystis* provides a high-quality food source for urchins^2, 53^. Purple sea urchins feeding on lower-quality diets have been shown to have significantly lower growth and feeding rates than those on higher-quality diets^54^, and the urchin barren urchins that we collected had particularly low gonad indices (a proxy for condition) that were ~ 11–12 times lower than the kelp forest urchins in our experiment. The poor condition of urchin barren urchins prior to the study, and their lower overall feeding rates relative to the kelp forest urchins during the experiment, thus likely led to their increased mortality.

Absolute differences between the grazing rates of kelp forest urchins and urchin barren urchins over time and across treatments are also unlikely to simply reflect differences in urchin condition. Differences in the nutritional quality between kelp and coralline algae are also likely to have affected consumption and digestion rates. Algal shapes or textures that increase handling time by herbivores can influence feeding rates^55, 56^, and the coralline algae we used in our study has articulated pinnate branching with cylindrical stipes, which may be more difficult for urchins to grasp, bite or scrape than broad, smooth, and flat bull kelp blades. Further, a diet of kelp has been shown to accelerate reproductive maturation and growth rate in sea urchins relative to a diet of coralline algae^57^, indicating its nutritional superiority. Regardless of the precise mechanism underlying the observed grazing rates, the proportional changes in grazing from the initial non-upwelling conditions to the first upwelling events were of similar magnitude for both kelp forest urchins and urchin barrens urchins, indicating that initial exposure to treatment conditions affected grazing rates similarly, regardless of food quality.

Despite that urchin grazing rates were lower when exposed to conditions that simulated upwelling overall, one nuance was the observed temporal effect, where urchin grazing increased during the second upwelling event compared to the first. This could be explained by urchins experiencing the first upwelling event as a physiological “shock” to which the urchins acclimated once the second upwelling event occurred (e.g.,^58^). In this regard, kelp forest urchins were also more resilient than urchin barren urchins, with their grazing rates rebounding to a larger degree under non-upwelling events. These differences in kelp forest and urchin barrens urchin performance highlight that environmental stress often imposes energetic trade-offs on organisms and that an organism’s condition, which is a product of their resource environment, will strongly influence how well it can balance those tradeoffs^50, 51^.

Although the integrated nature of our treatments did not allow us to precisely indicate which upwelling-related stressor had the largest effect on urchin grazing, the observed pattern of increased grazing during non-upwelling events and reduced grazing during upwelling events are consistent with previous work (e.g.,^18–20^). In general, it has been shown that increasing temperatures increase metabolic activity, thus leading to higher grazing rates in many organisms^59^. We hypothesize that increased temperatures during non-upwelling events led to higher grazing rates for both kelp forest and barren urchins during these times compared to upwelling events. The four degree increase from 12 °C during current day non-upwelling events to 16 °C in Future 2 non-upwelling events may also explain associated increases in urchin grazing between these treatments. Recent experimental work has shown that when purple urchins were exposed to low temperatures, low pH, and low DO separately, low temperature and DO had significant negative effects on respiration and grazing rates, but low pH did not; however, when combined, pH, temperature and DO can have interactive effects^19^. For example, low temperatures and low DO together had larger combined effects on urchin respiration and grazing than either did alone^19^. In contrast, when low DO or low temperature was combined with low pH, low pH seemed to lessen the effects of temperature and DO on urchin respiration and grazing^19^. When all three were combined, low pH was not enough to compensate for combined negative effects of low temperature and low DO^18, 20^. Because we saw a large reduction in grazing during upwelling events, it is reasonable to believe that low DO in our future treatments was mainly responsible for a decrease in grazing for both kelp forest urchins and urchin barren urchins. It should be noted, however, that our study reached low levels of pH and high temperatures for longer periods of time than in^19^. Further, much of what we simulate with our experimental treatments could also be considered marine heatwave conditions^60^. Specifically, some of our future “low” temperatures are higher than current low or high temperatures and are associated with low dissolved oxygen—such higher temperatures can exacerbate low oxygen effects by increasing metabolic rates. It is therefore possible that pH and temperature played direct or indirect roles in reducing urchin grazing here as well. Further studies independently altering pH and temperature to the levels reported here for a similar exposure time could elucidate the relative importance of each.

We also observed a decrease in gonad development in kelp forest urchins under distant future conditions (Future 3). Increased metabolism might explain the lower gonad indices in urchins in this treatment, as higher metabolic costs would leave less energy available for gonad production and energy storage. Metabolic rates increase with temperature across a wide range of organisms^59^ and increased metabolic costs associated with elevated *p*CO_2_ have been observed in sea urchins^61, 62^. Sea urchin reproductive processes may also be sensitive to higher-frequency patterns of variability in sublethal hypoxia. For example, sea urchins that spend longer periods of time in low oxygen conditions tend to produce smaller gonads than urchins under ambient oxygen conditions^20^. We therefore note that although we observed a significant decrease in gonad index in urchins under distant future conditions relative to other treatments, longer exposure to experimental conditions might have produced larger differences among treatments.

Although complexity in our experimental design did not allow us to isolate the effects of upwelling intensity vs. duration or the individual effects of stressors on urchins, our results are novel and important as individual stressors associated with upwelling are never independent in nature and upwelling intensity and duration are predicted to increase together in the future in some regions. Varying exposure to multiple upwelling stressors could have important implications for sea urchin population dynamics and kelp forest ecosystem structure. Fewer gonads in sea urchins reduce reproductive capacity^52, 63^, especially at lower urchin densities^64^, and gonads are a main target for predators due to their nutritional value^65^, so effects on gonad production could also reduce trophic energy transfer. Further, urchin grazing is critical to shallow subtidal ecosystems, and its effects could influence kelp forest dynamics and ‘tipping points’ between alternate community states^2, 66^. Therefore, present-day spatial differences in the intensity and duration of upwelling and future changes in upwelling exposure could have population- and ecosystem-level effects via direct impacts on one or two sensitive organism-level responses, such as urchin grazing and gonad development. However, because increased nutrient concentrations from upwelling and temperature increases from ocean warming can both directly affect kelp^67, 68^, understanding how future conditions will affect the kelp-urchin interaction will require determining whether upwelling-driven increases in nutrients will lead to increases in kelp growth that will surpass consumption by stressed urchin grazers^16^, and whether ocean warming with continue to deplete already-diminishing kelp forests^5, 69^. Further, future studies should incorporate how changes in upwelling duration and magnitude might affect early life stages of urchins, whose rapid growth may show differences in response to environmental stressors more quickly than adults^70, 71^.

As climate change alters the magnitude and duration of physiological stressors in ecosystems, subpopulations of organisms will be differentially affected^72, 73^ It is therefore necessary to examine the effects of different exposures under realistic patterns of variability, and how energetic context mediates these effects. We showed that exposure to increased intensity and duration of upwelling (as predicted by some climate models) has negative impacts on ecologically important purple sea urchins, and that these impacts are mediated by urchin diet and condition (a result of their habitat). Further, the responses of *S. purpuratus* to multiple stressors could not have been predicted from studies of single stressors, which would have produced results opposite to what we observed under conditions more likely to occur in the future (i.e., elevated temperature, hypoxia, and *p*CO_2_). This highlights the importance of understanding the combined effects of multiple stressors on marine organisms^74^ now, and in the future.

## Methods

### Urchin collection and condition

We collected urchins via SCUBA from urchin barrens at Baker Beach (41.03′N, 124.07′W), and kelp forests in Trinidad Harbor (41.07′N, 124.08′W) in northern California in July 2019. Urchins were transported to the Telonicher Marine Laboratory, weighed (blotted wet mass) and measured (test diameter). We used urchins averaging 43.23 ± 3.62 mm in test diameter because in nearby populations all individuals > 40 mm produce mature gametes with the ratio of gonad to body size peaking between 40 and 50 mm^75^. In urchin barrens, dense urchins and lack of kelp lead to poorly nourished individuals with reduced gonads relative to kelp forests, indicative of a food-mediated energetic tradeoff^52^. Thus, we chose urchins of this size to minimally detect habitat-specific differences and maximize potential for gonad development during our experiment. To ensure urchins from different habitats differed in condition, we collected *n* = 15 from each habitat (kelp forest: 42.88 ± 6.47 mm; urchin barrens: 43.87 ± 6.20 mm) and calculated gonad indices ((blotted wet mass gonad)/(total wet body mass) * 100), which are useful for comparing the condition of similarly-sized individuals in a population from different habitats through time^75^. Urchins were placed in individual chambers, starved for 24 h, and acclimated to experimental conditions for at least 24 h. Kelp forest urchins were fed bull kelp (*Nereocystis luetkeana*) and urchin barren urchins were fed articulated coralline algae (*Corallina* spp*.*), ad libitum.

### Experimental set-up and design

**Figure 5:**
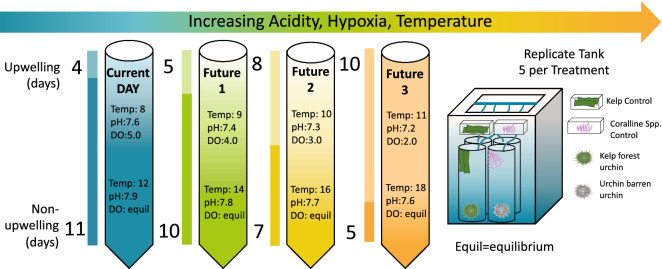
Schematic of experimental design and treatment description for the mesocosm experiment. Seawater chemistry parameters for each upwelling severity treatment are (L-R): reservoir 1—Current Day (blue), reservoir 2—Future 1 (green), reservoir 3—Future 2 (yellow), reservoir 4—Future 3 (orange). Both upwelling and non-upwelling event conditions regarding seawater parameters are listed in each corresponding reservoir, with upwelling on top and non-upwelling on the bottom. Colored bars located on the left side of each reservoir represent the duration of each upwelling and non-upwelling event in days. Lighter colors represent upwelling, and darker colors represent non-upwelling. Each reservoir delivered treated water to five randomly distributed replicate tanks. Each replicate tank housed four columns, each with one sea urchin, for a total of two kelp forest urchins and two urchin barren urchins (n = 10 urchins per treatment combination). The individual urchins in each column were completely separate from one another and could not interact physically, though the columns were perforated allowing treated water in each tank to intermingle among columns. See figure key for additional components of each replicate tank.

We established four upwelling severity treatments where we manipulated the duration of upwelling and non-upwelling events, and the magnitude of pH, temperature, and dissolved oxygen to represent current day and future-predicted conditions (Fig. 5). Treatments consisted of two upwelling and two non-upwelling events that alternated over a 30 day period. The proportion of upwelling days varied across treatments. In Treatment 1 (Current Day), urchins were exposed to 11 days of non-upwelling conditions, followed by exposure to 4 days of upwelling, with the sequence repeated once. Average current-day durations of upwelling events, and magnitudes of pH, DO, and temperature for upwelling and non-upwelling events were based on 2018 data from the CeNCOOS Trinidad shore station^29^. Future 1, 2, and 3 treatments were created by increasing the duration of upwelling events by 25, 100, and 150%, respectively; and by increasing upwelling temperature by 1 °C and decreasing upwelling pH and DO by 0.1 unit and 1.0 mg L^−1^, respectively, for each treatment. Non-upwelling temperatures were increased by 2 °C for each treatment. Non-upwelling DO was allowed to reach equilibrium with the atmosphere based on the given treatment temperature and pH. Increases in the duration of upwelling and the magnitudes of associated stressors were within range of historical patterns of change^28, 42^ and model predictions^30, 35–38, 42–44^.

Treatments were established in separate 208L insulated reservoirs, supplied with flow-through seawater and maintained via float valves. Treatments were replicated 5 times in 80L insulated tanks drawing from their corresponding reservoir. Each tank had four cylindrical, perforated PVC columns (41.4 cm × 15.2 cm) housing one urchin each, totaling two urchin barren and two kelp forest urchins per tank and 10 per treatment (Fig. 5). Each tank had a manifold controlling water flow to each column and assuring similar flows rates for urchins. To maintain treatments, reservoirs had two pumps; one for vertical mixing, and one circulating treated water to a manifold that diverted a fraction into each treatment tank manifold, with the remaining fraction circulated back to the reservoir. This partially-closed system stabilized treatment conditions between the reservoir and treatment tanks. Treatment tank water drained from the system, preventing chemical signals from the urchins passing among replicate tanks. When switching between upwelling and non-upwelling events, treatment tank conditions matched reservoirs within 120 min (Fig. 5).

To lower pH, CO_2_ was bubbled through 60 mm fine-pore air stones into each reservoir. Each reservoir had one integrated sensor (WTW pH 3310, Loligo Systems) through which pH and temperature were monitored and logged (Loligo Systems CapCTRL software) at 1 s intervals. Hysteresis for each pH treatment was set to ± 0.01 pH units, so if reservoir water deviated from the desired setpoint by 0.01, gas was added or stopped. pH probes were calibrated regularly using three-point calibration. DO was manipulated by bubbling pure N_2_ gas through 60 mm fine-pore diffusers. Each reservoir had one ocular DO probe (Vernier Software and Technology) controlled by an Arduino that monitored and recorded DO in mg L^−1^ every second. Temperature was controlled using aquarium chillers (JBJ Arctica Aquarium Chiller), heaters (titanium 1000-W) and heat pumps (DSHP-4 thru DSHP-6, Aqua Logic, Aquatic Design).

Discrete water samples were collected in 350 mL amber glass bottles roughly every week (n = 5–6/treatment) to verify treatment conditions and calculate carbonate chemistry. Samples were immediately poisoned with 100 µL of saturated mercuric chloride (HgCl_2_) and processed to measure *p*CO_2_ and DIC via gas equilibration and stripping, respectively; followed by infrared detection^76^ modified for discrete samples^77^. To ensure accuracy, gas and liquid standards included the complete range of values for ocean seawater, and the instrument was calibrated with Certified Reference Materials provided by A. Dickson (Scripps Inst. Oceanogr.). To calculate complete carbonate chemistry (including pH, total alkalinity [TA], Ω aragonite, and Ω calcite) we used the seacarb package in R^78, 79^.

### Urchin grazing

To assess whether grazing by kelp forest and urchin barren urchins were differentially affected by upwelling severity, we measured grazing rates throughout the experiment. To maintain the natural condition and diet of urchins from each habitat, kelp forest urchins were fed bull kelp and urchin barren urchins were fed coralline algae (*Corallina* spp*.*) ad libitum*.* Algae were fastened at the top of each column to force urchins to expend energy to graze and create a realistic scenario where urchins in declining kelp forests, where our urchins were sourced^5, 80^, have to shift from sheltering-in-place to active foraging, including climbing the stipes of kelp^6, 7^. To control for autogenic changes in kelp and coralline algae in the absence of urchins, a separate perforated container with each alga was placed within each replicate tank, which we used as our control algae. Both kelp and coralline algae were dried via manual centrifuge (spun 5 times) and weighed to the nearest 0.01 g before fed to urchins. To capture grazing differences between upwelling and non-upwelling events, we measured grazing rates (g algae day^−1^) every 3–4 days, as kelp degrades in the laboratory after ~ 4 days. The number of grazing rate measurements was proportional to the duration of each upwelling/non-upwelling event (e.g., if the non-upwelling event lasted 11 days and the upwelling event lasted 4 days, we took 3 measurements during the non-upwelling event, and 1 during the upwelling event). When multiple grazing rate measurements were taken per event, we averaged them to estimate grazing during the event. Final algal weights were obtained as with initial weights. To account for autogenic change, we subtracted the weight change in control algae from that in grazed algae.

### Urchin survival and gonad development

Urchins were measured for test diameter (mm) and total wet mass (g) at the beginning and end of the experiment. Throughout, we recorded urchin mortality and replaced dead urchins immediately, recording final diameter and mass. We dissected each urchin at the end of the experiment and calculated its gonad index. We used the subset of 30 urchins destructively sampled at the beginning of the experiment to obtain habitat-specific initial gonad indices, allowing us to calculate the difference in gonad indices due to experimental treatment effects.

### Statistical analysis

Residuals were checked for normality and homoscedasticity via Shapiro Wilk and Levene’s test, respectively. All data, except for mortality, met assumptions and so were not transformed. To assess differences in gonad wet mass between kelp forest and urchin barrens urchins we used ANCOVA with urchin habitat as a fixed factor and total wet mass as a covariate. Because urchin grazing rates were measured during each upwelling and non-upwelling event, and so were not independent, we used a mixed effects model with repeated measures to analyze them, with upwelling severity treatment, urchin habitat, time (upwelling event), and their interactions as fixed factors, and tank as a random factor. To compare across groups, including among interactions of factors, we used least square means separation tests, adjusting P-values for multiple comparisons with the Tukey method. We assessed differences in urchin mortality with a generalized linear mixed model with a Poisson family distribution and a log-link function with upwelling severity treatment and urchin habitat as fixed factors, and tank as a random factor. Because of the high mortality in urchin barren urchins, we only assessed treatment differences in gonad change for kelp forest urchins, using mixed model ANOVA with treatment as a fixed factor, and tank as a random factor. We used Tukey’s HSD test to make *post-hoc* comparisons among treatment means. Replacements for dead urchins were excluded from this analysis. All analyses were done in R^81^.

## Supplementary Information

Supplementary Information.

## References

[1] Krumhansl KA, Okamoto DK, Rassweiler A, Novak M, Bolton JJ, Cavanaugh KC, Connell SD, Johnson CR, Konar B, Ling SD, Micheli F, Norderhaug KM, Pérez-Matus A, Sousa-Pinto I, Reed DC, Salomon AK, Shears NT, Wernberg T, Anderson RJ, Barrett NS, Buschmann AH, Carr MH, Caselle JE, Derrien-Courtel S, Edgar GJ, Edwards M, Estes JA, Goodwin C, Kenner MC, Kushner DJ, Moy E, Nunn J, Steneck RS, Vásquez J, Watson J, Witman JD, Byrnes JE (2016). Global patterns of kelp forest change over the past half-century. Proc. Natl. Acad. Sci. USA.

[2] Filbee-Dexter K, Scheibling RE (2014). Sea urchin barrens as alternative stable states of collapsed kelp ecosystems. Mar. Ecol. Prog. Ser..

[3] Steneck RS, Graham MH, Bourque BJ, Corbett D, Erlandson JM, Estes JA, Tegner MJ (2002). Kelp forest ecosystems: biodiversity, stability, resilience and future. Environ. Conserv..

[4] Steneck RS (2020). Regular sea urchins as drivers of shallow benthic marine community structure. Dev. Aquacult. Fish. Sci..

[5] Rogers-Bennett L, Catton CA (2019). Marine heat wave and multiple stressors tip bull kelp forest to sea urchin barrens. Sci. Rep..

[6] Pearse JS (2006). Ecological role of purple sea urchins. Science.

[7] Harrold C, Reed DC (1985). Food availability, sea urchin grazing, and kelp forest community structure. Ecology.

[8] Kriegisch N, Reeves SE, Flukes EB, Johnson CR, Ling SD (2019). Drift-kelp suppresses foraging movement of overgrazing sea urchins. Oecologia.

[9] Pearse JS, Hines AH (1987). Long-term population dynamics of sea urchins in a central California kelp forest: rare recruitment and rapid decline. Mar. Ecol. Prog. Ser..

[10] Watanabe JM, Harrold C (1991). Destructive grazing by sea urchins *Strongylocentrotus* spp. in a central California kelp forest: potential roles of recruitment, depth, and predation. Mar. Ecol. Prog. Ser..

[11] Reid J, Rogers-Bennett L, Vasquez F, Pace M, Catton CA, Kashiwada JV, Taniguchi IK (2016). The economic value of the recreational red abalone fishery in northern California. Calif. Fish Game.

[12] Menge BA, Menge DN (2013). Dynamics of coastal meta-ecosystems: the intermittent upwelling hypothesis and a test in rocky intertidal regions. Ecol. Monogr..

[13] Breitburg DL, Loher T, Pacey CA, Gerstein A (1997). Varying effects of low dissolved oxygen on trophic interactions in an estuarine food web. Ecol. Monogr..

[14] Hauri C, Gruber N, Plattner GK, Alin S, Feely RA, Hales B, Wheeler PA (2009). (2009) Ocean acidification in the California current system. Oceanography.

[15] Connell SD, Russell BD (2010). The direct effects of increasing CO_2_ and temperature on non-calcifying organisms: increasing the potential for phase shifts in kelp forests. Proc. R. Soc. B.

[16] Sellers, A. J. *et al*. Seasonal upwelling reduces herbivore control of tropical rocky intertidal algal communities. *Ecology* e03335 10.1002/ecy.3335(2021). 10.1002/ecy.3335 33709403

[17] Moulin L, Grosjean P, Leblud J, Batigny A, Dubois P (2014). Impact of elevated *p*CO_2_ on acid-base regulation of the sea urchin *Echinometra mathaei* and its relation to resistance to ocean acidification: a study in mesocosms. J. Exp. Mar. Biol. Ecol..

[18] Siikavuopio SI, Dale T, Mortensen A, Foss A (2007). Effects of hypoxia on feed intake and gonad growth in the green sea urchin, *Strongylocentrotus droebachiensis*. Aquaculture.

[19] Low, H. N. N. *The Effects of Upwelling-driven Hypoxia on Sea Urchins in California Current Kelp Forests*. PhD dissertation, Stanford University, Stanford, CA (2018).

[20] Low NH, Micheli F (2018). Lethal and functional thresholds of hypoxia in two key benthic grazers. Mar. Ecol. Prog. Ser..

[21] Low NH, Micheli F (2020). Short-and long-term impacts of variable hypoxia exposures on kelp forest sea urchins. Sci. Rep..

[22] Huyer A (1983). Coastal upwelling in the California current system. Prog. Oceanogr..

[23] Frieder, C. A., Nam, S. H., Martz, T. R. & Levin, L. A. High temporal and spatial variability of dissolved oxygen and pH in a nearshore California kelp forest. *Biogeosciences* **9**, 3917–3930 (2012).

[24] Feely RA, Okazaki RR, Cai WJ, Bednaršek N, Alin SR, Byrne RH, Fassbender A (2018). The combined effects of acidification and hypoxia on pH and aragonite saturation in the coastal waters of the California current ecosystem and the northern Gulf of Mexico. Cont. Shelf Res..

[25] Chan F, Barth JA, Blanchette CA, Byrne RH, Chavez F, Cheriton O, Feely RA, Friederich G, Gaylord B, Gouhier T, Hacker S, Hill T, Hofmann G, McManus MA, Menge BA, Nielsen KJ, Sanford E, Sevadjian J, Washburn L (2017). Persistent spatial structuring of coastal ocean acidification in the California Current System. Sci. Rep..

[26] Orr JC, Fabry VJ, Aumont O, Bopp L, Doney SC, Feely RA, Gnanadesikan A, Gruber N, Ishida A, Joos F, Key RM, Lindsay K, Maier-Reimer E, Matear R, Monfray P, Mouchet A, Najjar RG, Plattner G-K, Rodgers KB, Sabine CL, Sarmiento JL, Schlitzer R, Slater RD, Totterdell IJ, Weirig M-F, Yamanaka Y, Yool A (2005). Anthropogenic ocean acidification over the twenty-first century and its impact on calcifying organisms. Nature.

[27] Feely RA, Alin SR, Carter B, Bednaršek N, Hales B, Chan F, Sabine CL (2016). Chemical and biological impacts of ocean acidification along the west coast of North America. Estuar. Coast. Shelf Sci..

[28] Iles AC, Gouhier TC, Menge BA, Stewart JS, Haupt AJ, Lynch MC (2012). Climate-driven trends and ecological implications of event-scale upwelling in the California Current *System*. Glob. Change Biol..

[29] CeNCOOS. *Real-Time Sensor Feeds of Oceanographic and Atmospheric Models’ Online Tool to Extract Temperature, pH, and Dissolved Oxygen*. https://data.cencoos.org (2020).

[30] Bakun A (1990). Global climate change and intensification of coastal ocean upwelling. Science.

[31] McGregor HV, Dima M, Fischer HW, Mulitza S (2007). Rapid 20th-century increase in coastal upwelling off northwest Africa. Science.

[32] Narayan N, Paul A, Mulitza S, Schulz M (2010). Trends in coastal upwelling intensity during the late 20th century. Ocean Sci..

[33] Barton EDD, Field DBB, Roy C (2013). Canary current upwelling: more or less?. Prog. Oceanogr..

[34] Mote PW, Mantua NJ (2002). Coastal upwelling in a warmer future. Geophys. Res. Lett..

[35] Bakun A, Black BA, Bograd SJ, Garcia-Reyes M, Miller AJ, Rykaczewski RR, Sydeman WJ (2015). Anticipated effects of climate change on coastal upwelling ecosystems. Curr. Clim. Change Rep..

[36] Wang D, Gouhier TC, Menge BA, Ganguly AR (2015). Intensification and spatial homogenization of coastal upwelling under climate change. Nature.

[37] Snyder, M. A., Sloan, L. C., Diffenbaugh, N. S. & Bell, J. L. Future climate change and upwelling in the California Current. *Geophys. Res. Lett.* **30**, 1823 (2003).

[38] García‐Reyes, M. & Largier, J. Observations of increased wind‐driven coastal upwelling off central California. *J. Geophys. Res. Oceans* **115**, 1–8 (2010).

[39] Varela, R., Álvarez, I., Santos, F., DeCastro, M. & Gómez-Gesteira, M. Has upwelling strengthened along worldwide coasts over 1982–2010?. *Sci. Rep.* **5**, 1–15 (2015). 10.1038/srep10016 PMC4424801 25952477

[40] Varela, R., Lima, F. P., Seabra, R., Meneghesso, C. & Gómez-Gesteira, M. Coastal warming and wind-driven upwelling: a global analysis. *Sci. Total Environ.* **639**, 1501–1511 (2018). 10.1016/j.scitotenv.2018.05.273 29929313

[41] Abrahams A, Schlegel RW, Smit AJ (2021). Variation and change of upwelling dynamics detected in the world’s eastern boundary upwelling systems. Front. Mar. Sci..

[42] Pachauri RK, Meyer LA, IPCC, Core Writing Team (2014). Climate change 2014: Synthesis report. Contribution of Working Groups I, II and III to the Fifth Assessment Report of the Intergovernmental Panel on Climate Change.

[43] Rykaczewski, R. R. & Dunne, J. P. Enhanced nutrient supply to the California Current Ecosystem with global warming and increased stratification in an earth system model. *Geophys. Res. Lett.* **37**, 1-5 (2010).

[44] Somero GN, Beers JM, Chan F, Hill TM, Klinger T, Litvin SY (2016). What changes in the carbonate system, oxygen, and temperature portend for the northeastern Pacific Ocean: a physiological perspective. Bioscience.

[45] Frölicher TL, Fischer EM, Gruber N (2018). Marine heatwaves under global warming. Nature.

[46] Filbee-Dexter K, Wernberg T, Grace SP, Thormar J, Fredriksen S, Narvaez CN, Feehan CJ, Norderhaug KM (2020). Marine heatwaves and the collapse of marginal North Atlantic kelp forests. Sci. Rep..

[47] Sokolova IM, Frederich M, Bagwe R, Lannig G, Sukhotin AA (2012). Energy homeostasis as an integrative tool for assessing limits of environmental stress tolerance in aquatic invertebrates. Mar. Environ. Res..

[48] Sokolova IM (2013). Energy-limited tolerance to stress as a conceptual framework to integrate the effects of multiple stressors. Integr. Comp. Biol..

[49] Fitzgerald-Dehoog L, Browning J, Allen BJ (2012). Food and heat stress in the California mussel: evidence for an energetic trade-off between survival and growth. Biol. Bull..

[50] Ramajo L, Pérez-León E, Hendriks IE, Marbà N, Krause-Jensen D, Sejr MK, Blicher ME, Lagos NA, Olsen YS, Duarte CM (2016). Food supply confers calcifiers resistance to ocean acidification. Sci. Rep..

[51] Brown NE, Bernhardt JR, Anderson KM, Harley CD (2018). Increased food supply mitigates ocean acidification effects on calcification but exacerbates effects on growth. Sci. Rep..

[52] Wahle RA, Peckham SH (1999). Density-related reproductive trade-offs in the green sea urchin,* Strongylocentrotus droebachiensis*. Mar. Biol..

[53] Rogers-Bennett L, Allen BL, Rothaus DP (2011). Status and habitat associations of the threatened northern abalone: importance of kelp and coralline algae. Aquat. Conserv. Mar. Freshw. Ecosyst..

[54] Brown MB, Edwards MS, Kim KY (2014). Effects of climate change on the physiology of giant kelp, *Macrocystis pyrifera*, and grazing by purple urchin, *Strongylocentrotus purpuratus*. Algae.

[55] Klinger TS, Lawrence JM (1985). Distance perception of food and the effect of food quantity on feeding behavior of *Lytechinus variegatus* (Lamarck) (Echinodermata: Echinoidea). Mar. Freshw. Behav. Physiol..

[56] Trowbridge CD (1995). Establishment of the green alga *Codium fragile* ssp. *tomentosoides* on New Zealand rocky shores: current distribution and invertebrate grazers. J. Ecol..

[57] Meidel SK, Scheibling RE (1999). Effects of food type and ration on reproductive maturation and growth of the sea urchin *Strongylocentrotus droebachiensis*. Mar. Biol..

[58] Harianto J, Nguyen HD, Holmes SP, Byrne M (2018). The effect of warming on mortality, metabolic rate, heat-shock protein response and gonad growth in thermally acclimated sea urchins (*Heliocidaris erythrogramma*). Mar. Biol..

[59] Brown JH, Gillooly JF, Allen AP, Savage VM, West GB (2004). Toward a metabolic theory of ecology. Ecology.

[60] Hobday AJ, Alexander LV, Perkins SE, Smale DA, Straub SC, Oliver EC, Benthuysen JA, Burrows MT, Donat MG, Feng M, Holbrook NJ, Moore PJ, Scannell HA, Gupta AS, Wernberg T (2016). A hierarchical approach to defining marine heatwaves. Prog. Oceanogr..

[61] Spicer JI, Widdicombe S, Needham HR, Berge JA (2011). Impact of CO_2_-acidified seawater on the extracellular acid-base balance of the northern sea urchin *Strongylocentrotus dröebachiensis*. J. Exp. Mar. Biol. Ecol..

[62] Catarino AI, Bauwens M, Dubois P (2012). Acid–base balance and metabolic response of the sea urchin *Paracentrotus lividus* to different seawater pH and temperatures. Environ. Sci. Pollut. Res..

[63] Rogers-Bennett L, Bennett WA, Fastenau HC, Dewees CM (1995). Spatial variation in red sea urchin reproduction and morphology: implications for harvest refugia. Ecol. Appl..

[64] Quinn JF, Wing SR, Botsford LW (1993). Harvest refugia in marine invertebrate fisheries: models and applications to the red sea urchin, *Strongylocentrotus franciscanus*. Am. Zool..

[65] Eurich JG, Selden RL, Warner RR (2014). California spiny lobster preference for urchins from kelp forests: implications for urchin barren persistence. Mar. Ecol. Prog. Ser..

[66] Steneck RS, Leland A, McNaught DC, Vavrinec J (2013). Ecosystem flips, locks, and feedbacks: the lasting effects of fisheries on Maine's kelp forest ecosystem. Bull. Mar. Sci..

[67] Gerard VA (1982). Growth and utilization of internal nitrogen reserves by the giant kelp *Macrocystis pyrifera* in a low-nitrogen environment. Mar. Biol..

[68] Simonson EJ, Scheibling RE, Metaxas A (2015). Kelp in hot water: I. Warming seawater temperature induces weakening and loss of kelp tissue. Mar. Ecol. Prog. Ser..

[69] Thomsen MS, Mondardini L, Alestra T, Gerrity S, Tait L, South PM, Lilley SA, Schiel DR (2019). Local extinction of bull kelp (Durvillaea spp.) due to a marine heatwave. Front. Mar. Sci..

[70] O’Donnell MJ, Hammond LM, Hofmann GE (2009). Predicted impact of ocean acidification on a marine invertebrate: elevated CO_2_ alters response to thermal stress in sea urchin larvae. Mar. Biol..

[71] Dupont S, Dorey N, Stumpp M, Melzner F, Thorndyke M (2013). Long-term and trans-life-cycle effects of exposure to ocean acidification in the green sea urchin Strongylocentrotus droebachiensis. Mar. Biol..

[72] Marcel EV, Adriaensen F, Van Balen JH, Blondel J, Dhondt AA, Van Dongen S, Matthysen E (2003). Variable responses to large-scale climate change in European *Parus* populations. Proc. R. Soc. B.

[73] Parker LM, Ross PM, O’Connor WA (2011). Populations of the Sydney rock oyster, *Saccostrea glomerata*, vary in response to ocean acidification. Mar. Biol..

[74] Kroeker KJ, Kordas RL, Harley CD (2017). Embracing interactions in ocean acidification research: confronting multiple stressor scenarios and context dependence. Biol. Lett..

[75] Conor JJ (1972). Gonad growth in the sea urchin, *Strongylocentrotus purpuratus* (Stimpson) (Echinodermata: Echinoidea) and the assumptions of gonad index methods. J. Exp. Mar. Biol. Ecol..

[76] Bandstra L, Hales B, Takahashi T (2006). High-frequency measurements of total CO_2_: method development and first oceanographic observations. Mar. Chem..

[77] Hales B, Chipman D, Takahashi T (2004). High-frequency measurement of partial pressure and total concentration of carbon dioxide in seawater using microporous hydrophobic membrane contactors. Limnol. Oceanogr. Methods.

[78] Lavigne, H., Epitalon, J. M. & Gattuso, J. P. *Seacarb: Seawater Carbonate Chemistry with R*. R package version 3.0 http://CRAN.R-project.org/package=seacarb (2011).

[79] Gattuso, J. P., Epitalon, J. M., Lavigne, H. & Orr, J. *Seacarb: seawater carbonate chemistry*. R package version 3.2.10. http://CRAN.R-project.org/package=seacarb (2018).

[80] Murie KA, Bourdeau PE (2020). Fragmented kelp forest canopies retain their ability to alter local seawater chemistry. Sci. Rep..

[81] R Core Team. *R: A Language and Environment for Statistical Computing*. R Foundation for Statistical Computing, Vienna, Austria. http://www.R-project.org/ (2013).

