# Supplementary material for: Energetic context determines the effects of multiple upwelling-associated stressors on sea urchin performance
Source: Sci Rep. 2021 May 31;11:11313. doi: 10.1038/s41598-021-90608-6 (PMC8167166; doi:10.1038/s41598-021-90608-6)
Supplement: Supplementary file 1 — Supplementary Information. [file 41598_2021_90608_MOESM1_ESM.docx]

**Supplementary information**

**Title:** Energetic context determines the effects of multiple upwelling-associated stressors on sea urchin performance.

**Authors:** Kindall A. Murie^1,2*^ & Paul E. Bourdeau^1,2^

**Author affiliations:** ^1^Telonicher Marine Laboratory, Humboldt State University, Trinidad, USA.

^2^Department of Biological Sciences, Humboldt State University, Arcata, USA.

^*^**email and current address:** [kmurie@uw.edu](mailto:kmurie@uw.edu), Department of Biology, University of Washington, Seattle, USA

Supplementary Fig. S1. Relationship between total wet mass (g) and gonad wet mass (g) for a subset of kelp forest (green symbols and line) and urchin barren (purple symbols and line) urchins (n=15 for each) used to determine average initial gonad index for urchins used in our experiment. Lines are ordinary least-squares fit. *R*^2^_kelp forest urchins_ = 0.40, *R*^2^_urchin barren urchins_ = 0.56).

Supplementary Fig. S2. Hourly average (A) pH, (B) temperature, and (C) dissolved oxygen in each of the four upwelling severity treatment reservoirs over the duration of the mesocosm experiment.

| Supplementary Table1. Post-hoc mean separation tests for predicted marginal means for (a) all factor combinations in the linear model, and averaged over the levels of (b) urchin condition and (c) upwelling severity treatment. Letters denote groups that are not significantly different from one another. Significance level used α = 0.05. The Sidak and Tukey methods were used for confidence-level and *P*-value adjustment, respectively. | | | | | | | | |
| --- | --- | --- | --- | --- | --- | --- | --- | --- |
| (a) Habitat x Treatment x Time | | | | | | | | |
| Habitat | Treatment | Time | ls mean | SE | df | lower CI | upper CI | group |
| Kelp Forest | Future 2 | 2 | -1.47 | 0.34 | 16 | -2.76 | -0.19 | a |
| Kelp Forest | Future 2 | 4 | -0.15 | 0.34 | 16 | -1.44 | 1.14 | abc |
| Kelp Forest | Future 3 | 4 | -0.13 | 0.25 | 16 | -1.09 | 0.83 | ab |
| Kelp Forest | Current Day | 2 | -0.11 | 0.49 | 19 | -1.91 | 1.70 | abc |
| Urchin Barren | Current Day | 2 | -0.06 | 0.49 | 19 | -1.87 | 1.74 | abcd |
| Urchin Barren | Future 1 | 2 | -0.02 | 0.34 | 16 | -1.31 | 1.26 | abc |
| Urchin Barren | Future 3 | 4 | 0.00 | 0.25 | 16 | -0.96 | 0.96 | abc |
| Urchin Barren | Future 2 | 4 | 0.13 | 0.34 | 16 | -1.16 | 1.42 | abce |
| Urchin Barren | Future 3 | 2 | 0.15 | 0.25 | 16 | -0.82 | 1.11 | abc |
| Urchin Barren | Future 2 | 2 | 0.17 | 0.34 | 16 | -1.12 | 1.45 | abce |
| Urchin Barren | Future 2 | 3 | 0.29 | 0.34 | 16 | -0.99 | 1.59 | bce |
| Urchin Barren | Future 3 | 1 | 0.32 | 0.47 | 16 | -1.45 | 2.09 | abcdefg |
| Urchin Barren | Future 3 | 3 | 0.34 | 0.34 | 16 | -0.94 | 1.63 | abce |
| Urchin Barren | Current Day | 4 | 0.52 | 0.47 | 19 | -1.20 | 2.24 | abcdef |
| Urchin Barren | Future 1 | 3 | 0.59 | 0.34 | 16 | -0.70 | 1.88 | abce |
| Urchin Barren | Current Day | 3 | 0.59 | 0.29 | 19 | -0.46 | 1.64 | bcd |
| Kelp Forest | Future 3 | 2 | 0.62 | 0.25 | 16 | -0.35 | 1.58 | bce |
| Urchin Barren | Future 2 | 1 | 0.63 | 0.34 | 16 | -0.66 | 1.92 | bcdef |
| Urchin Barren | Current Day | 1 | 0.70 | 0.29 | 19 | -0.36 | 1.74 | bcdef |
| Urchin Barren | Future 1 | 4 | 0.81 | 0.29 | 16 | -0.27 | 1.89 | bce |
| Urchin Barren | Future 1 | 1 | 0.86 | 0.29 | 16 | -0.22 | 1.94 | bce |
| Kelp Forest | Future 1 | 2 | 1.06 | 0.34 | 16 | -0.23 | 2.35 | bcdefgh |
| Kelp Forest | Future 3 | 1 | 1.15 | 0.47 | 16 | -0.62 | 2.92 | bcdefghij |
| Kelp Forest | Future 1 | 1 | 1.47 | 0.29 | 16 | 0.39 | 2.55 | bcdefghi |
| Kelp Forest | Current Day | 1 | 2.05 | 0.29 | 19 | 1.00 | 3.10 | efghij |
| Kelp Forest | Current Day | 4 | 2.28 | 0.47 | 19 | 0.56 | 3.10 | cdefghijk |
| Kelp Forest | Future 1 | 3 | 2.59 | 0.34 | 16 | 1.30 | 3.88 | dfghijk |
| Kelp Forest | Current Day | 3 | 2.76 | 0.29 | 19 | 1.71 | 3.81 | ghijk |
| Kelp Forest | Future 2 | 1 | 3.15 | 0.34 | 16 | 1.86 | 4.43 | hijk |
| Kelp Forest | Future 1 | 4 | 3.16 | 0.29 | 16 | 2.09 | 4.25 | jk |
| Kelp Forest | Future 2 | 3 | 3.33 | 0.34 | 16 | 2.04 | 4.62 | ijk |
| Kelp Forest | Future 3 | 3 | 4.27 | 0.34 | 16 | 2.98 | 5.56 | k |
|  |  |  |  |  |  |  |  |  |
| (b) Treatment x Time | |  |  |  |  |  |  |  |
| Habitat | Treatment | Time | ls mean | SE | df | lower CI | upper CI | group |
| -- | Future 2 | 2 | -0.65 | 0.25 | 16 | -1.53 | 0.23 | a |
| -- | Current Day | 2 | -0.09 | 0.36 | 19 | -1.29 | 1.12 | abcd |
| -- | Future 3 | 4 | -0.06 | 0.20 | 16 | -0.75 | 0.62 | ab |
| -- | Future 2 | 4 | -0.01 | 0.25 | 16 | -0.89 | 0.87 | abc |
| -- | Future 3 | 2 | 0.38 | 0.20 | 16 | -0.30 | 1.06 | abcde |
| -- | Future 1 | 2 | 0.52 | 0.25 | 16 | -0.36 | 1.40 | abcdef |
| -- | Future 3 | 1 | 0.73 | 0.34 | 16 | -0.44 | 1.91 | abcdefg |
| -- | Future 1 | 1 | 1.16 | 0.22 | 16 | 0.41 | 1.92 | cdefgh |
| -- | Current Day | 1 | 1.38 | 0.22 | 19 | 0.64 | 2.11 | efgh |
| -- | Current Day | 4 | 1.40 | 0.34 | 19 | 0.25 | 2.55 | bcdefgh |
| -- | Future 1 | 3 | 1.59 | 0.25 | 16 | 0.71 | 2.47 | defgh |
| -- | Current Day | 3 | 1.68 | 0.22 | 19 | 0.94 | 2.41 | fgh |
| -- | Future 2 | 3 | 1.81 | 0.25 | 16 | 0.93 | 2.69 | fgh |
| -- | Future 2 | 1 | 1.89 | 0.25 | 16 | 1.01 | 2.76 | fgh |
| -- | Future 1 | 4 | 1.99 | 0.22 | 16 | 1.23 | 2.74 | gh |
| -- | Future 3 | 3 | 2.31 | 0.25 | 16 | 1.43 | 3.19 | h |
|  |  |  |  |  |  |  |  |  |
| (c) Habitat x Time | |  |  |  |  |  |  |  |
| Habitat | Treatment | Time | ls mean | SE | df | lower CI | upper CI | group |
| Kelp Forest | -- | 2 | 0.02 | 0.18 | 16 | -0.55 | 0.60 | a |
| Urchin Barren | -- | 2 | 0.06 | 0.18 | 16 | -0.52 | 0.63 | a |
| Urchin Barren | -- | 4 | 0.37 | 0.17 | 16 | -0.18 | 0.91 | a |
| Urchin Barren | -- | 3 | 0.45 | 0.16 | 16 | -0.06 | 0.97 | a |
| Urchin Barren | -- | 1 | 0.63 | 0.18 | 16 | 0.07 | 1.18 | ab |
| Kelp Forest | -- | 4 | 1.29 | 0.17 | 16 | 0.75 | 1.84 | bc |
| Kelp Forest | -- | 1 | 1.95 | 0.18 | 16 | 1.40 | 2.51 | c |
| Kelp Forest | -- | 3 | 3.24 | 0.16 | 16 | 2.72 | 3.75 | d |
